# Chronic alcohol-induced long-lasting working memory deficits are associated with altered histone H3K9 dimethylation in the prefrontal cortex

**DOI:** 10.3389/fnbeh.2024.1354390

**Published:** 2024-03-01

**Authors:** Mael De Clerck, Martin Manguin, Nadia Henkous, Marion N. d’Almeida, Daniel Beracochea, Nicole Mons

**Affiliations:** INCIA, CNRS-UMR5287, Bâtiment Bordeaux Biologie Santé, Bordeaux, France

**Keywords:** chronic alcohol, histone methylation, memory, prefrontal cortex, epigenetics, mice

## Abstract

**Introduction:**

Epigenetic modifications have emerged as key contributors to the enduring behavioral, molecular and epigenetic neuroadaptations during withdrawal from chronic alcohol exposure. The present study investigated the long-term consequences of chronic alcohol exposure on spatial working memory (WM) and associated changes of transcriptionally repressive histone H3 lysine 9 dimethylation (H3K9me^2^) in the prefrontal cortex (PFC).

**Methods:**

Male C57BL/6 mice were allowed free access to either 12% (v/v) ethanol for 5 months followed by a 3-week abstinence period or water. Spatial WM was assessed through the spontaneous alternation T-maze test. Alcoholic and water mice received daily injections of GABAB agonist baclofen or saline during alcohol fading and early withdrawal. Global levels of histone modifications were determined by immunohistochemistry.

**Results:**

Withdrawal mice displayed WM impairments along with reduced prefrontal H3K9me^2^ levels, compared to water-drinking mice. The withdrawal-induced decrease of H3K9me^2^ occurred concomitantly with increased level of permissive H3K9 acetylation (H3K9ac) in the PFC. Baclofen treatment rescued withdrawal-related WM deficits and fully restored prefrontal H3K9me^2^ and H3K9ac. Alcohol withdrawal induced brain region-specific changes of H3K9me^2^ and H3K9ac after testing, with significant decreases of both histone marks in the dorsal hippocampus and no changes in the amygdala and dorsal striatum. Furthermore, the magnitude of H3K9me^2^ in the PFC, but not the hippocampus, significantly and positively correlated with individual WM performances. No correlation was observed between H3K9ac and behavioral performance. Results also indicate that pre-testing intraperitoneal injection of UNC0642, a selective inhibitor of histone methyltransferase G9a responsible for H3K9me^2^, led to WM impairments in water-drinking and withdrawal-baclofen mice. Collectively, our results demonstrate that alcohol withdrawal induced brain-region specific alterations of H3K9me^2^ and H3K9ac, an effect that persisted for at least three weeks after cessation of chronic alcohol intake.

**Conclusion:**

The findings suggest a role for long-lasting decreased H3K9me^2^ specifically in the PFC in the persistent WM impairments related to alcohol withdrawal.

## 1 Introduction

The cessation of alcohol after chronic alcohol consumption (CAC) leads to the formation of long-lasting changes in brain function that mediate the myriad symptoms associated with withdrawal, including anxiety disorders and cognitive deficits. Clinical and experimental studies in humans and rodents have provided evidence to suggest that alteration of the functional integrity of the medial prefrontal cortex (PFC) is a key factor underlying long-lasting cognitive and behavioral deficits during the withdrawal period following CAC (Abernathy et al., 2010; Kroener et al., 2012; Fowler et al., 2014; Trantham-Davidson et al., 2017). Rodents exposed to prolonged alcohol intake showed persistent deficits in spatial working memory (WM) tasks, which rely on interactions between the PFC and the dorsal hippocampus (dHPC) (Funahashi, 2017; Wirt and Hyman, 2017). Using a mouse model of a 5-month CAC, we previously reported that C57BL/6 mice display sustained deficits in spatial WM on a T-maze task (Béracochéa and Jaffard, 1987; Dominguez et al., 2016, 2017). These deficits persisted up to six weeks after ethanol withdrawal and were associated with sustained elevation of corticosterone concentration in the PFC (Dominguez et al., 2016, 2017, 2018) and with persistent decreases of cAMP-CREB pathway and histone H4 acetylation within the PFC (Pascual et al., 2011; Dominguez et al., 2016).

A prime mechanism that could underlie the alcohol-induced memory impairments involves long-term changes in brain gene expression through post-translational modifications of histones, both in humans and animal models (Pandey et al., 2001; Qiang et al., 2011). Notably, brain region-specific changes of G9a, a key histone methyltransferase (HMT) responsible for dimethylation of Lysine 9 on histone H3 (H3K9me^2^), a molecular marker associated with transcriptional silencing, has recently emerged as important contributor to different alcohol-related behaviors, including withdrawal-related anxiety, alcohol tolerance and dependence and in alcohol-related neurodevelopmental disorders (Benevento et al., 2015). Chronic intermittent ethanol exposure reduced both G9a and H3K9me2 in the nucleus accumbens which was associated with a decrease of stress-regulated alcohol drinking in male mice (Anderson et al., 2022). Further, significant downregulation of G9a activity and decreased H3K9 methylation have been implicated in the persistent ethanol-induced neuroadaptation of NR2B gene following chronic intermittent ethanol treatment using either cortical neuronal cell culture or mice *in vivo* (Qiang et al., 2011). Other studies have shown that increased G9a-mediated H3K9me2 in the amygdala produced rapid tolerance to the anxiolytic properties of ethanol, which was reversed by pharmacological G9a inhibition in adults rats (Schaefer et al., 2009; Berkel et al., 2018; Wang et al., 2018). Conversely, systemic administration of the G9a inhibitor UNC0642 to mice *in utero* has the opposite effect, resulting in increased anxiety-related behaviors and decreased social interaction in adulthood. Furthermore, studies have shown an essential role for G9a-mediated H3K9me^2^ mechanisms in regulating the expression of genes that are important for alcohol use and/or stress (Maze et al., 2010, 2014).

The present study was designed to test the hypothesis that withdrawal from chronic alcohol exposure would result in persistent WM impairments and lead to corresponding long-lasting, decrease in the repressive histone mark H3K9me^2^ in the PFC and related regions. By using mice exposed to a 5-month CAC followed by a 3-week withdrawal period, we first investigated the effects of alcohol withdrawal on spatial WM and global levels of H3K9me^2^ in the PFC. We previously showed that treatment with baclofen, an agonist of GABA-B receptor, during early withdrawal alleviates ethanol seeking behavior and glucocorticoid dysfunction as well as contextual memory deficits (Rabat et al., 2019; Henkous et al., 2022). Therefore, we examined the consequences of baclofen treatment on WM and regional patterns of H3K9me^2^ in water and withdrawal mice. Finally, we evaluated the effects of an acute systemic injection of the G9a inhibitor UNC0642 on WM performance in withdrawal-baclofen and water mice.

## 2 Materials and methods

### 2.1 Animals

Male C57BL/6 mice (10-month-old) were used throughout the experiments. Upon arrival at 4-month-old, they were group-housed with food and water/alcohol solutions provided *ad libitum*, under standard conditions (22 ± 1°C); 12-h light–dark cycle (light on at 7 h00). Two weeks before the experiments, they were single-housed and handled daily 5 min/day to avoid non-specific stress to the future experiments. All experimental procedures were conducted between 9:00–12:00 a.m. to prevent any circadian rhythm side effects. Procedures were performed in accordance with the local ethical committee (CEE50, approval # 24077) and were conducted according to the European Union Directive 2010/63/European Union guidelines for animal experiments.

### 2.2 Chronic alcohol administration and withdrawal

**Figure 1:**
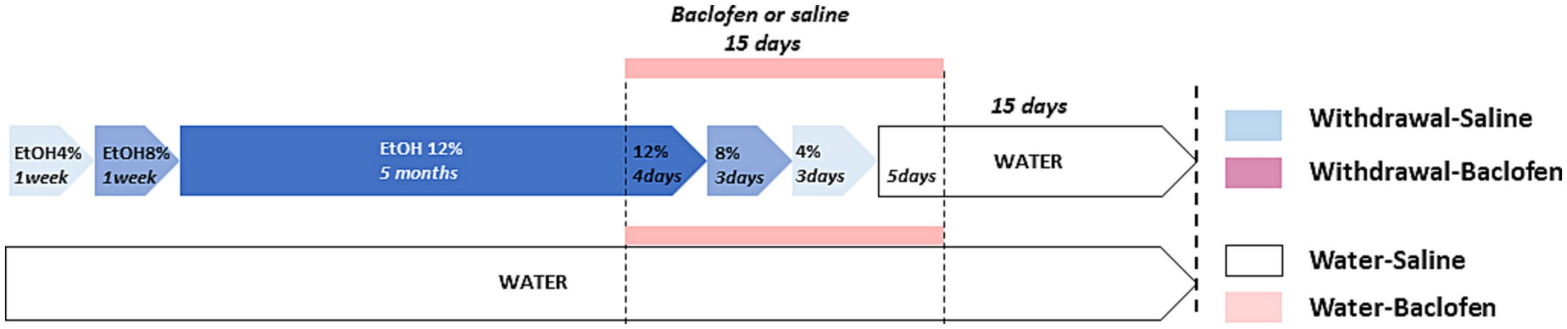
Schematic design of the four groups of mice used for behavioral, immunohistochemical and pharmacological analyses. The withdrawal mice were given increasing doses of ethanol (4 and 8% v/v for 1 week each; then 12% v/v for 5 months). During the withdrawal phase, mice received daily IP injection of baclofen (Withdrawal-Baclofen) or saline (Withdrawal-Saline) during the 15-day withdrawal period (12% for 4 days; 8% for 3 days; 4% for 3 days; water for 5 days). The Water-Saline and Water-Baclofen mice were given water as a sole source of fluid and received daily IP saline or baclofen injections during the 15-day withdrawal period. Behavioral testing began 2 weeks after the last injection of baclofen or saline.

The C57BL/6 inbred mouse strain is known for its strong preference for alcohol over water (Mulligan et al., 2008). Four-month-old mice were given as their sole liquid source an ethanol (Prochilab, France) solution varying by the alcohol concentration as follows: 4% (v/v) for a week, 8% (v/v) for a 2nd week, and then 12% (v/v) for 5 months. At the end of CAC, they were assigned to withdrawal condition consisting of a series of deescalating ethanol concentration: 12% for 4 days, 8% for 3 days, 4% for 3 days before given *ad libitum* access to water for 2 weeks until behavioral testing. Experimental design is schematized in Figure 1. Solutions were freely available to mice in two 250 mL bottles in each cage. The fact that animals were housed in collective cages (*N* = 10/cage) did not allow individual measures of alcohol consumption. We have previously reported that the mean daily alcohol intake was 4.2 mL/mouse, namely 8.57 g/kg/day of alcohol (Dominguez et al., 2017). The daily alcohol intake during alcohol exposure in the different cages was similar: hence, we may legitimately infer that all mice were equally exposed to alcohol, thus allowing for valuable comparisons among the different cohorts. The water animals had access to water during CAC and withdrawal periods.

### 2.3 Drug treatments

Baclofen (Liorésal®, Novartis) was diluted in saline solution (0.9% NaCl, pH 7.4) and injected intraperitoneally (IP) (10 mL/kg, 1 injection/day) during the 15 days of alcohol fading phase and early withdrawal. Baclofen was progressively decreased to avoid potential negative effects of an abrupt drug cessation (1.5 mg/kg for Days 15 to 4; 0.75 mg/kg for Days 3–2; 0.37 mg/kg for Day 1). Water and withdrawal mice were randomly assigned into two groups before being IP injected with either baclofen (Withdrawal-Baclofen and Water-Baclofen) or saline (Withdrawal-Saline and Water-Saline) solution. Behavioral testing began at least 2 weeks after the last injection that is to say at a time at which baclofen was not detectable in blood (Rabat et al., 2019).

The G9a HMT-inhibitor UNC0642 (G9a_inh_) (Sigma-Aldrich) was dissolved in 0.1 mol/L sodium citrate buffer (pH 7.4) at a concentration of 4 mg/Kg, 2 mg/kg and 1 mg/kg, (Berkel et al., 2018) and preliminary data. Water-Saline and Withdrawal-Baclofen mice were IP injected with G9a_inh_ or vehicle 30 min before behavioral testing [Water-Saline: vehicle (*N* = 10), UNC-1 (*N* = 8), UNC-2 (*N* = 7) or UNC-4 (*N* = 9); Withdrawal-Baclofen: vehicle (*N* = 10), UNC-1 (*N* = 6); UNC-2 (*N* = 9) or UNC-4 (*N* = 7)].

### 2.4 Behavioral task

The sequential alternation task in the T-maze is commonly used to assess WM in rodents. Behavioral tests were conducted in a grey Plexiglas T-maze. Stem and arms are 35 cm long, 15 cm wide and 10 cm high. The starting box (10 cm × 12 cm) and each goal-arm were separated from the central alley by a vertical sliding door, with opening and closing monitored by a controller. The T-maze was located at the center of a room with various allocentric cues (white, black or striped card boards) located on the wall.

The task is based on the innate tendency of rodents to explore the two goal-arms of the maze over successive trials (Dominguez et al., 2016). Indeed, to alternate from trial to trial, the mouse is required to remember at a given N trial the choice made at the N_−1_ trial, and reset the interfering information from previous trials (N_−2_, N_−3_…). Proactive interferences are therefore determined by the progressive fall of alternation rate in the sequential procedure, which depends both on the number of trials in the series and on the delay separating trials. According to previous data (Béracochéa and Jaffard, 1987; Vandesquille et al., 2013), training and testing sessions consisted of a series of 7 successive trials separated by a constant inter-trial interval (ITI) for training (30 s) and testing (90 s).

#### 2.4.1 Training

The four groups of mice (*N* = 9 for each group) were first habituated to the T-maze and allocentric cues during two 10 min-free exploration sessions occurring on 2 successive days. They were then subjected to a training phase (7 successive trials separated by a 30 s-ITI delay) to familiarize them with the experimental procedure (opening and closing of doors and confinement into the arms). Typically, for each trial, after a 30 s confinement period in the start box, the mouse was allowed to enter in one of the goal-arms, confined for 30 s in the goal-box, and then, placed back to the start-box for a new trial. An alternation response was scored each time the subject entered the arm opposite to the one visited on the immediate trial N_−1_. To avoid olfactory cues in the apparatus, visible traces of urine and feces were washed with water.

#### 2.4.2 Testing

All mice were submitted 24 h later to the same procedure but with a 90 s-ITI delay. Previously, we have shown that this delay produces an exaggerated sensitivity to interference during the series in withdrawal mice (Dominguez et al., 2016). The mean alternation rate was calculated on the 7 successive trials and expressed in percentage (number of alternation/(number of trials-1) × 100). In order to analyze proactive interference effects within the test session, the session was divided in two Blocks of 3 consecutive trials and the mean alternation rate was calculated for Block A (trials 2–4) and Block B (trials 5–7). Running latencies were registered, allowing calculation of the mean choice latency ± SEM (in sec). To dissociate memory deficit from an possible progressive loss of motivation to alternate over the series, an 8th trial separated by a shorter ITI (5 s) from the 7th one, was added. Indeed, shortening the ITI should restore alternation performance in experimental mice, ruling out therefore any motivational alterations as a causal factor of their WM deficits.

### 2.5 Immunochemistry and quantification

The mice (*N* = 9/group) were killed 30 min after the beginning of WM testing. Matched naive controls (*N* = 5/group) left undisturbed in their home-cage for the period of training and testing, were killed at the same times as their respective testing counterparts. All mice were deeply anesthetized with IP injection of ketamine (100 mg/kg)/xylazine (10 mg/kg) cocktail (Bayer, Wuppertal, Germany) and perfused with 100 mL of 4% paraformaldehyde in 0.1 M phosphate buffer (PB; pH 7.5). The brains were quickly removed, post-fixed in the same fixative overnight, sectioned into 40 μm-thick coronal sections using a vibratome (Leica) and then stored at −20°C in antifreeze solution until processed for immunohistochemistry. For immunodetection, free-floating sections were washed with Tris buffer saline (TBS, 0.1 M, pH 7.4) at 4°C for 3×15 min. After inhibition of the endogenous peroxidase activity with TBS containing 1% hydrogen peroxide and 10% methanol, and incubation in a saturation solution (TBS; 0.075% Tween; 8% goat serum), the sections were incubated for 48 h at 4°C in TBS containing polyclonal rabbit anti-H3K9me^2^ and rabbit anti-H3K9ac antibodies (1/3000, Millipore). Afterwards, the sections were then incubated with the biotinylated secondary anti-rabbit antibodies (1/2000 in TBS, Jackson Immunoresearch) for 2 h at room temperature. They were then washed and incubated with 2% avidin-biotin-peroxidase complex (ABC Elite kit, Vector Laboratories) for 90 min. The peroxidase reaction was developed in TB containing 2.5% diaminobenzidine tetrahydrochloride and 0.02% hydrogen peroxide. The color reaction was washed 2X15mn with TB and then with PB. Afterwards, the sections were mounted on gelatin-coated slides, dried and coverslipped with Eukitt. Images were captured using CDD video color Sony camera mounted on a BX-50 Olympus (10 × magnification). The regions of interest (PFC (prelimbic cortex)), area CA1 of the dHPC (dCA1), basolateral nucleus of the amygdala (AMG) and dorsal striatum were delineated using the atlas of (Paxinos and Franklin, 2001). The cell count was performed manually in in each side of 3–4 sections per animal through Image J software (ImageJ®) and the mean number of immunopositive nuclei/mm^2^ was calculated. The experimenter was blinded to experimental groups during counting. The mean number of cells for each brain area of the tested groups was normalized by mean values of respective control home-caged group.

### 2.6 Statistical analysis

Statistical analyses were performed using the Statview 5.0 software®. Data were expressed as mean ± SEM. The T-maze data were non-normally distributed. Behavioral data were therefore analyzed using Mann–Whitney tests for comparisons between groups. One-sample sign test was used to determine if performances were above chance levels (with hypothesized mean-chance level: 50% for correct responses). For correlation analyses, the Spearman’s correlation coefficient R was determined and a probability level of *p* < 0.05 was accepted as statistically significant. Immunohistochemistry and pharmacological data were analyzed using one-way or two-way ANOVA (Statview® 5.0 software). Post-hoc Bonferroni/Dunnett’s multiple comparisons analyses were performed when appropriate. Significance was set to *p* ≤ 0.05.

## 3 Results

### 3.1 Long-lasting WM deficits during withdrawal from chronic alcohol

**Figure 2:**
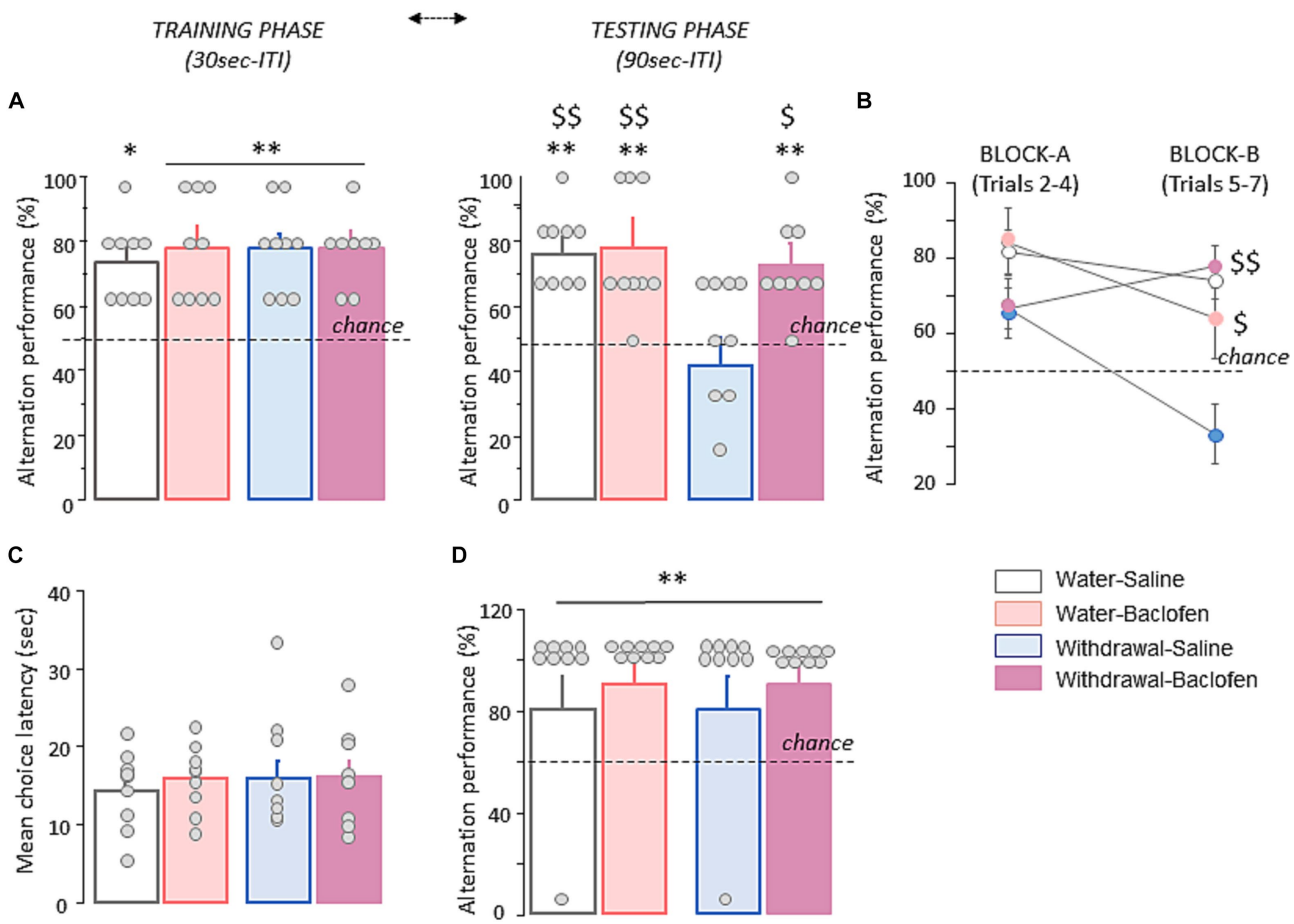
Alcohol withdrawal after chronic alcohol exposure leads to long-lasting WM deficits that are rescued by baclofen treatment during the withdrawal period. **(A)** Alternation performance is expressed as mean + SEM percentage of correct responses during the training *(left)* and testing *(right)* phases with 30s and 90s inter-trial intervals (ITI), respectively. **(B)** Alternation performance is expressed as mean + SEM percentage of correct responses during Block A (trials 2–4) and Block B (trials 5–7). **(C)** Mean choice latency expressed in sec during Trials 2–7. **(D)** Mean + SEM percentage of correct responses during trial 8 with a 5 s ITI. Dashed lines for A and B represent chance level. The asterisk and the dollar signs in the quantification graphs indicate significance using Mann–Whitney *U* test. **p < 0.01 and *p < 0.05 versus chance. ^$$^*p* < 0.01 and ^$^*p* < 0.05 versus Withdrawal-Saline. Bars represent mean + SEM.

Spatial WM was evaluated in four cohorts of mice (Water-Saline and Water-baclofen, Withdrawal-Saline and Withdrawal-Baclofen) through the spontaneous alternation T-maze test. During the training phase with a 30s ITI, all groups of mice alternated significantly above chance level (50%; all *p* < 0.01 except Water-baclofen: *p* < 0.05) at approximatively the same rate (between 73.3 and 77.8%; Figure 2A, *left*). During WM testing with a 90s ITI, the Water-Saline (77.80 ± 3.90%), Water-Baclofen (75.90 ± 6.3%) and Withdrawal-Baclofen (72.20 ± 4.8%) groups still performed well above chance level (*p* < 0.01 in all comparisons; Figure 2A, *right*). In contrast, the Withdrawal-Saline mice performed at chance (50.00 ± 6.20%) and had significantly lower correct responses compared to Water-Saline and Water-Baclofen mice (Mann–Whitney *U* = 8, *p* = 0.0041 and *U* = 15, *p* = 0.02; respectively). Baclofen treatment reversed the withdrawal-induced WM deficits (Withdrawal-Baclofen versus Withdrawal-Saline: *U* = 15, *p* = 0.024) restoring T-maze performance to control levels. An analysis per block (Block A: trials 2–4 and block B: trials 5–6) further revealed that Withdrawal-Saline mice were impaired on both blocks (versus chance: both *p* > 0.1) and significantly differed from Water-Baclofen (*U* = 16.5, *p* = 0.034), Water-Saline (*U* = 7, *p* = 0.0031) or Withdrawal-Baclofen (*U* = 6, *p* = 0.0023); Figure 2B). As shown in Figure 2C, the analysis of the mean choice latencies over trials indicated no between-group difference (Water-Saline: 14.074 ± 1.69 s; Water-Baclofen: 15.88 ± 1.44 s; Withdrawal-Baclofen: 16.18 ± 2.08 s; Withdrawal-Saline: 17.55 ± 2.46 s). Finally, all mice alternated similarly and significantly above chance level when tested on trial 8 with a 5 s ITI (*p* < 0.01 in all comparisons; Figure 2D), suggesting that withdrawal-induced WM deficits did not result from motivational or motor skills impairments. Taking together, our results confirmed the exaggerated sensitivity of Withdrawal-Saline mice to interference generated by successive trials with a 90s ITI and indicated that baclofen treatment during the withdrawal period was effective at preventing WM deficits induced by alcohol withdrawal.

### 3.2 Withdrawal-induced WM impairments are associated with decreased histone H3K9me^2^ in the PFC

**Figure 3:**
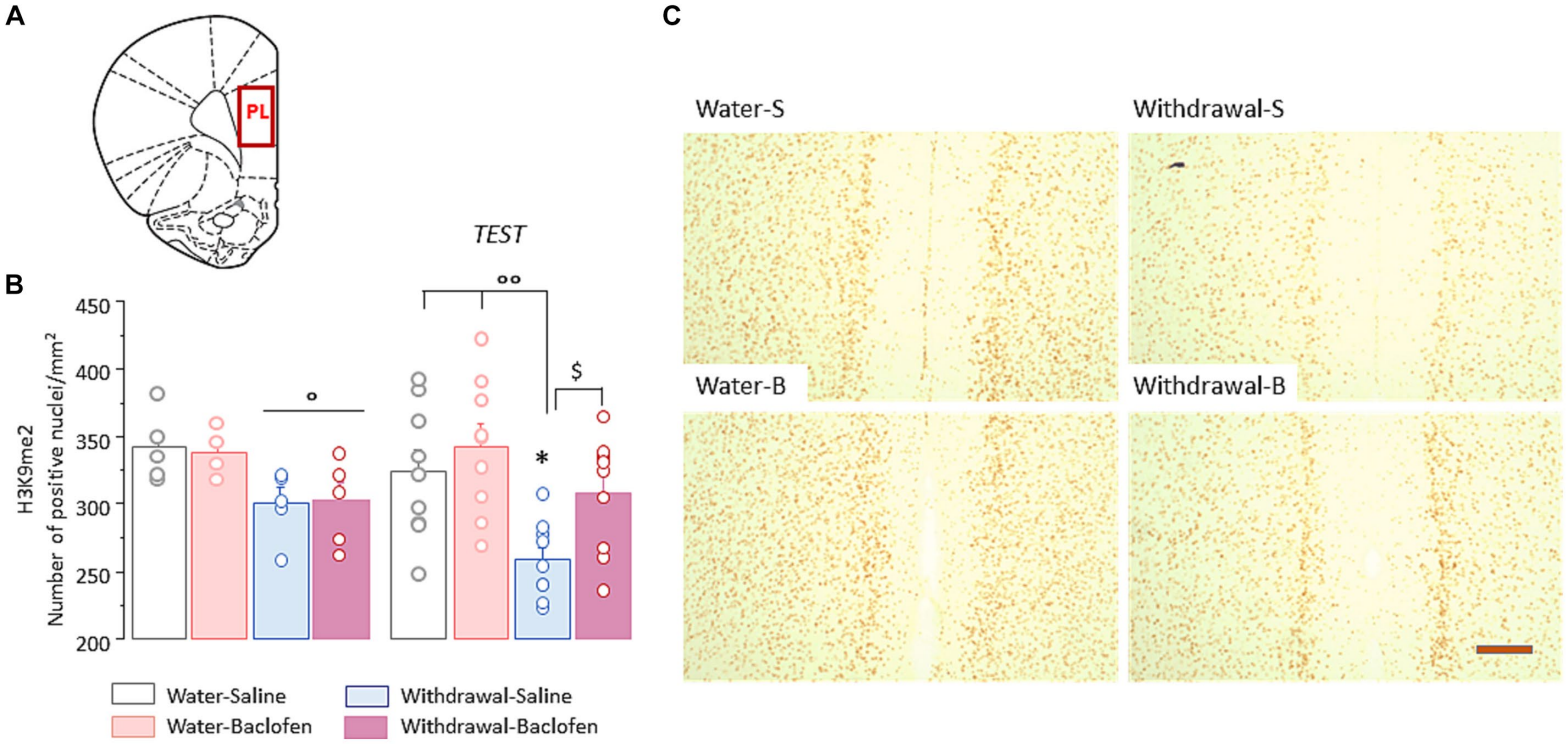
Prefrontal H3K9me^2^ levels are reduced in WM-impaired withdrawal-saline mice. **(A)** Schematic coronal section illustrating where the quantification of H3K9me^2^ and images were taken in the prefrontal cortex (PL). **(B)** Quantification of H3K9me^2^ in the PFC in Water and Withdrawal mice treated with Saline or baclofen and sacrificed before and 30 min after testing. Results are expressed as mean number of positive nuclei per mm^2^ + SEM. **(C)** Representative photomicrographs showing the testing-related changes of H3K9me^2^ immunoreactivities for Water-Saline and Water-Baclofen mice *(left)*, Withdrawal-Saline and Withdrawal-Baclofen *(right)* mice. Scale bar: 100 μm. *p < 0.05 versus respective Naïve; ^$^*p* < 0.05 versus Withdrawal-Baclofen; °°*p* < 0.01 and °*p* < 0.05 versus Water.

We then examined the changes of H3K9me^2^ in the PFC (Figure 3A) in the four cohorts of mice sacrificed 30 min after WM testing (Test, *N* = 9/group), compared with those of matched animals killed directly from their home cage (Naïve, *N* = 5/group).

The two-way ANOVA indicated a significant effect of withdrawal [*F*_(1,48)_ = 14.49, *p* < 0.001] but no effect of test and no interaction between withdrawal and test (both *F* < 1, both *p* > 0.1). Under naïve condition, withdrawal mice had significantly lower numbers of prefrontal H3K9me2 nuclei relative to water mice, irrespective of the saline or baclofen pretreatment (both *p* < 0.05; Figure 3A, left). After WM testing, Withdrawal-Saline mice showed further decrease in number of positive-H3K9me^2^ (versus Naïve: *p* < 0.05) while no changes were observed in other groups. As shown in Figure 3B right, Withdrawal-Saline group significantly differed from the two Water groups (both *p* < 0.01) and from the Withdrawal-Baclofen group (*p* < 0.05). Representative images of prefrontal H3K9me^2^ immunoreactivities after WM testing in the four groups are shown in Figure 3C.

### 3.3 Prefrontal histone H3K9ac is deregulated during alcohol withdrawal

**Figure 4:**
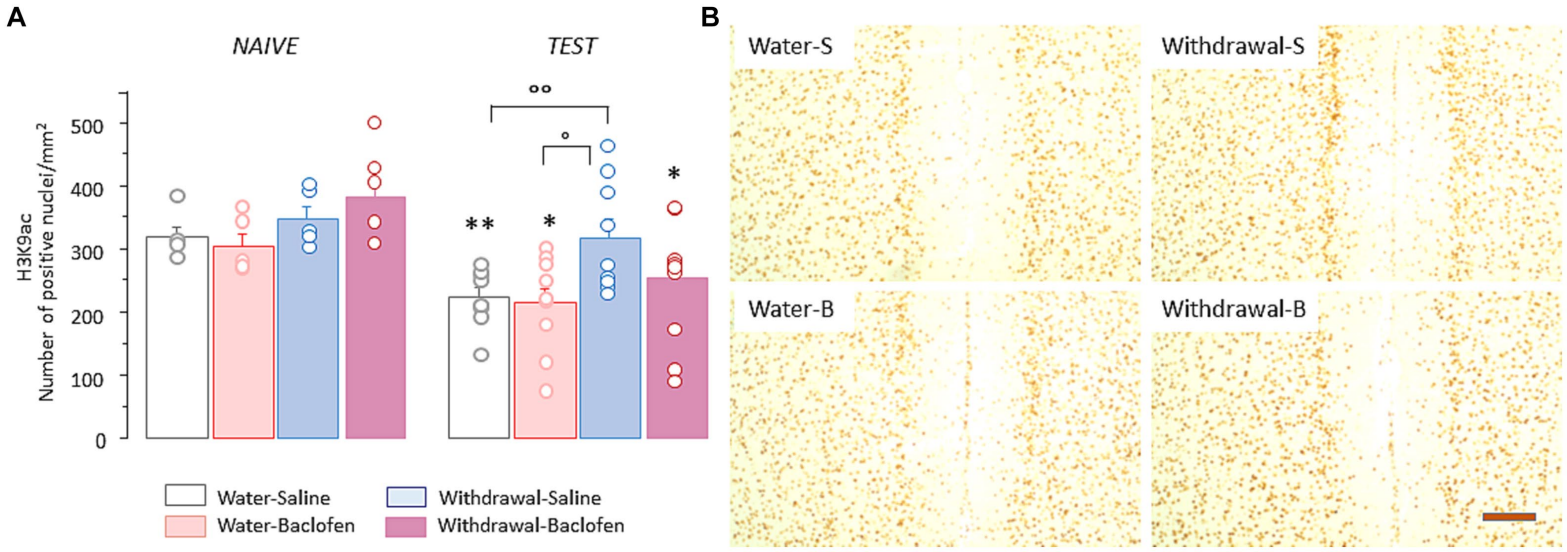
Prefrontal H3K9ac levels remain unchanged in WM-impaired Withdrawal-saline mice. **(A)** Quantification of H3K9ac in Water and Withdrawal mice treated with Saline or baclofen and sacrificed before and after testing. Results are expressed as mean number of positive nuclei per mm^2^ + SEM. **(B)** Representative photomicrographs showing the testing-related changes of H3K9ac immunoreactivities for Water-Saline and Water-Baclofen mice *(left)*, Withdrawal-Saline and Withdrawal-Baclofen mice *(right)*. Scale bar: 100 μm. **p < 0.01 and *p < 0.05 versus respective Naïve; °°*p* < 0.01 and °*p* < 0.05 versus Water.

Since H3K9 residue can be either acetylated or methylated, we also examined the effects of prolonged alcohol withdrawal on H3K9 acetylation (H3K9ac) in the PFC. Two-way ANOVA indicated significant effects of withdrawal [*F*_(1,48)_ = 8.46, *p* = 0.0053] and test [*F*_(1,48)_ = 17.5, *p* < 0.0001] without interaction between withdrawal and test [*F*_(1,48)_ =0.12, NS]. As shown in Figure 4A left, there was no between-group differences under naïve condition whereas WM testing induced significantly lower numbers of prefrontal H3K9ac in all groups except Withdrawal-Saline group (versus Naïve: Water-Saline: *p* < 0.01, Water-Baclofen and Withdrawal-Baclofen: both *p*s < 0.05). As shown in Figure 4A right, significant differences were found between Withdrawal-Saline and Water-Saline (*p* < 0.05) or Water-Baclofen (*p* < 0.01) groups. Representative images of prefrontal H3K9me^2^ immunoreactivities in the four groups after testing are shown in Figure 4B.

### 3.4 Changes of prefrontal H3K9me^2^ are positively correlated with working memory performance

**Figure 5:**
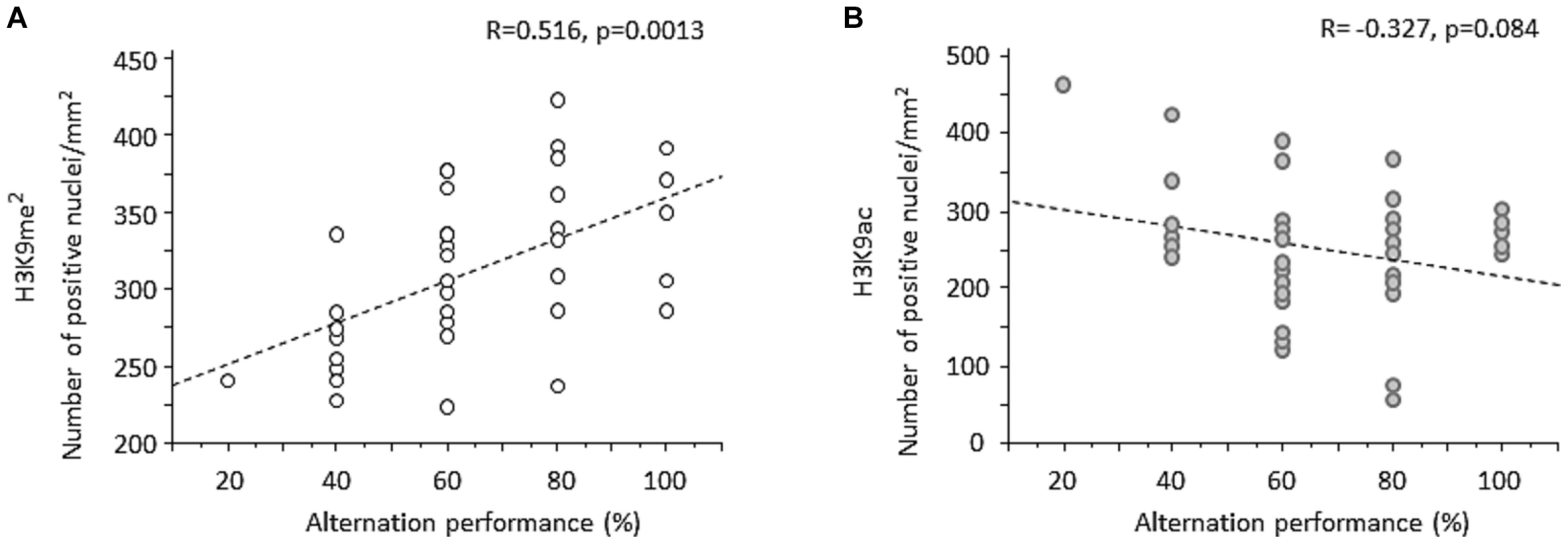
Regression analyses between spontaneous alternation performance and individual changes of H3K9me^2^ or H3K9ac in the PFC. **(A)** When data from all groups of mice were pooled together, individual prefrontal H3K9me^2^ levels were significantly positively correlated with percent alternation rates. **(B)** A negative non-significant correlation was found between individual H3K9ac and percent alternation rates.

Correlation analyses were applied to examine relationship between individual WM performance and the magnitude of prefrontal H3K9me^2^. Overall, when data from all groups of mice were pooled together, a strong positive correlation was found between individual percent alternation rates and changes of H3K9me^2^ (*R* = 0.516, *p* = 0.0013; Figure 5A). Examining the relationship between WM performance and H3K9me^2^ among each group confirmed strong positive correlations for Water-Saline (*R* = 0.872, *p* = 0.0047) and Withdrawal-Saline (*R* = 0.849, *p* = 0.0076) mice. The same analysis conducted on H3K9ac data indicated a non-significant negative correlation between H3K9ac and individual alternation performance (*R* = −0.327, *p* > 0.05; Figure 5B).

### 3.5 Withdrawal-saline mice display distinct patterns of H3K9me^2^ and H3K9ac in other brain regions

**Figure 6:**
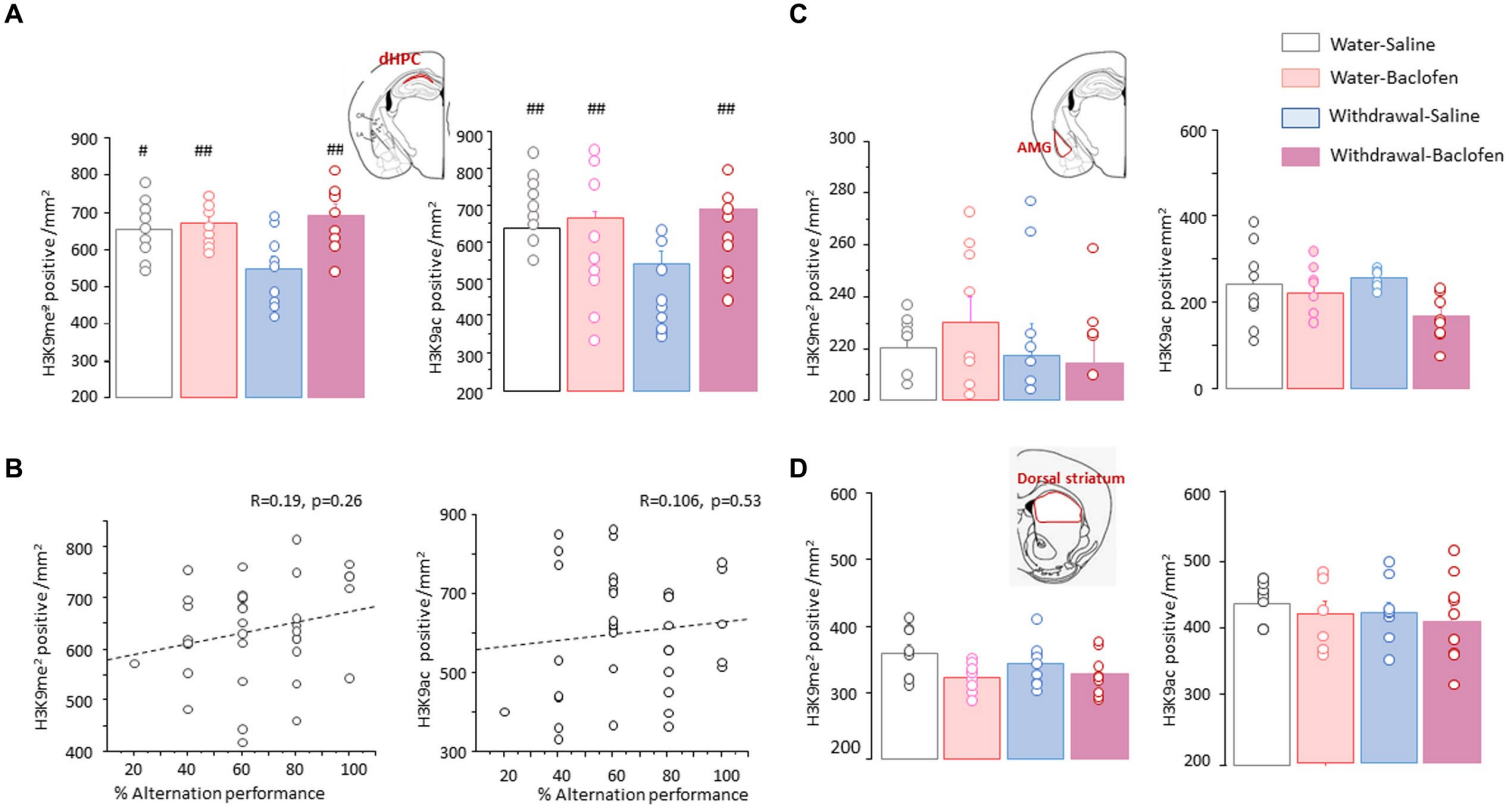
Effects of alcohol withdrawal on the testing-related changes of H3K9me2 and H3K9ac in the dCA1, amygdala and dorsal striatum. **(A)** Top Schematic drawing of area CA1 (*in red*) in the dorsal hippocampus (dHPC). Quantification of H3K9me^2^ *(left)* and H3K9ac *(right)* in Water and Withdrawal mice treated with Saline or baclofen and sacrificed 30 min after testing. **(B)** Regression analyses between individual alternation scores and the magnitude of H3K9me^2^ *(left)* or H3K9ac *(right)* in the dCA1 when data from the four groups of mice were pooled together. **(C,D)**. Top Schematic drawings *(in red)* and testing-related changes of H3K9me2 *(left)* and H3K9ac *(right)* in the basolateral amygdala [AMG, **(C)**] and the dorsal part of the striatum **(D)**. Results are expressed as mean number of positive nuclei per mm^2^ + SEM. ^##^*p* < 0.01 and ^#^*p* < 0.05 versus other groups.

To test the specificity of the effects observed in the PFC, we measured the numbers of H3K9me^2^- and H3K9ac-positive nuclei in area CA1 of the dHPC (dCA1), basolateral amygdala nucleus (AMG) and dorsal part of the striatum. The two-way ANOVA conducted on H3K9me^2^ or H3K9ac data in the dCA1 yielded significant effects for Test [*F*_(1,48)_ = 16.52; *p* < 0.001 and *F*_(1,48)_ = 20.04; *p* < 0.0001, respectively] and Group [*F*_(3,48)_ = 7.74; *p* < 0.001 and *F*_(3,48)_ =13.83; *p* < 0.0001, respectively], without interaction between Test X Group (both *F*_(3,48)_<1; NS). As shown in Figure 6A, Withdrawal-Saline mice displayed significantly reduced numbers of H3K9me^2^ (Figure 6A, left) and H3K9ac (Figure 6A, right) after testing compared to Water-Saline (*p* < 0.05 and *p* < 0.01, respectively), Water-Baclofen and Withdrawal-Baclofen mice (*p* < 0.01 in all comparisons). We performed correlation analysis between the magnitude of H3K9me^2^ or H3K9ac data in the dCA1 and individual alternation performance. When data from all groups of mice were pooled together, no statistically reliable correlation was observed between WM and H3K9me^2^ (*R* = 0.19; *p* = 0.26; Figure 6B, left) or H3K9ac (*R* = 0.106; *p* = 0.53; Figure 6B, right) in the dCA1. We also measured H3K9me^2^ and H3K9ac in the amygdala (Figure 6C) and the dorsal striatum (Figure 6D). Unlike the PFC and the dCA1, there was no evidence of any difference between Water and Withdrawal groups for H3K9me^2^ (Figures 6C,D, left) and H3K9ac (Figures 6C,D, right) in these regions.

### 3.6 Administration of the G9a inhibitor UNC0642 before testing disrupts WM performances in water-saline and withdrawal-baclofen mice

**Figure 7:**
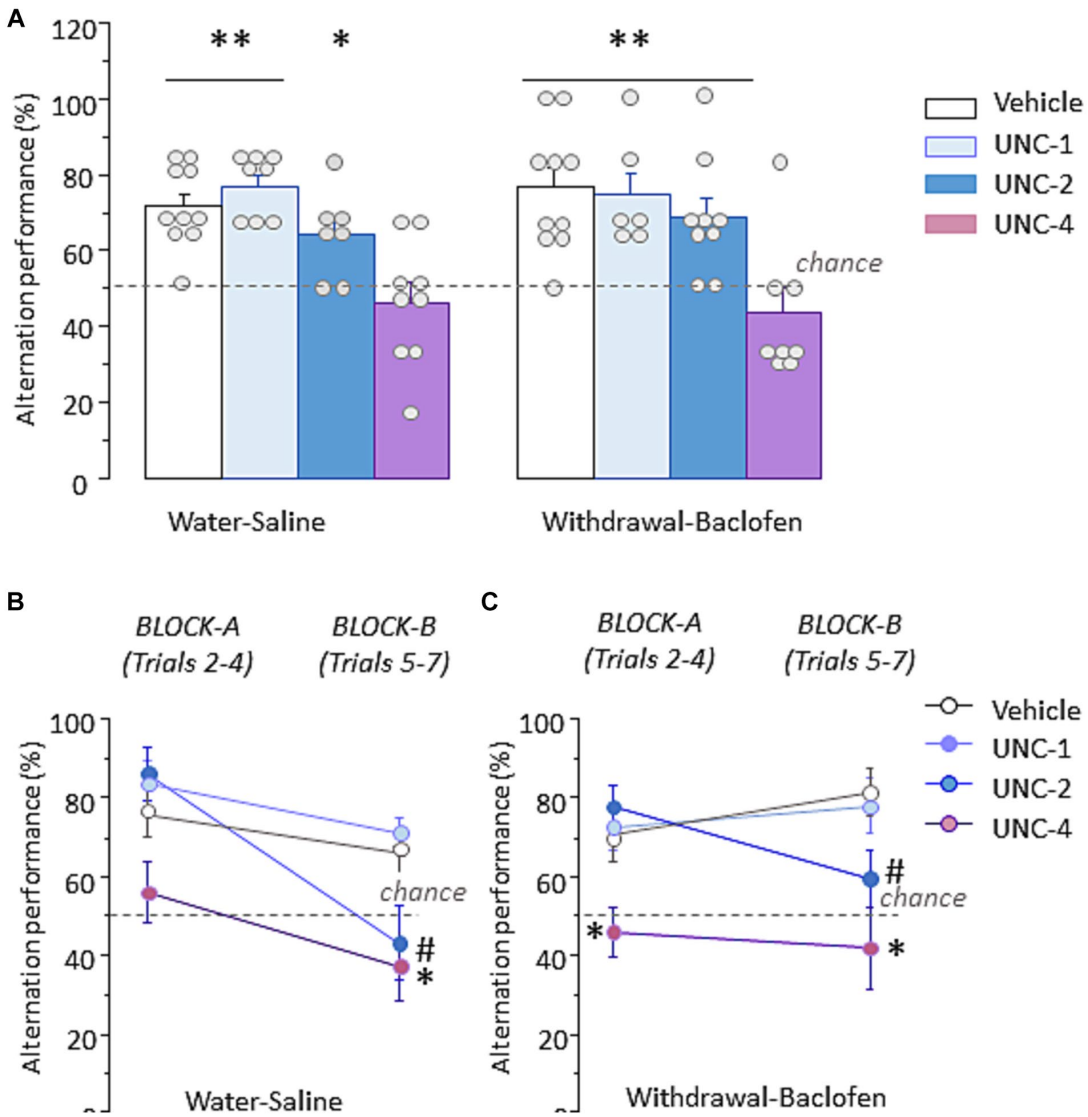
Pre-testing inhibition of the H3K9 methyltransferase G9a produces WM impairments in Water-Saline and Withdrawal-Baclofen mice. **(A)** Alternation performance expressed as mean + SEM percentage of correct responses during WM testing in Water-Saline *(left)* and withdrawal-Baclofen *(right)* mice injected with the G9a inhibitor UNC0642 at 1 mg/kg (UNC-1), 2 mg/kg (UNC-2) and 4 mg/kg (UNC-4) or with vehicle 30 min before testing. **(B,C)** Mean percentage of correct responses during Block A (trials 2–4) and Block B (trials 5–7) for Water-Saline **(B)** and Withdrawal-Baclofen **(C)**. Dashed lines represent chance level. **p < 0.01 and *p < 0.05 UNC-4 versus others; # UNC-2 versus vehicle or UNC-1.

To determine whether inhibition of the HMT G9a, a key histone methyltransferase responsible for H3K9 dimethylation, disrupts WM performance, three different doses (1 mg/kg: UNC-1, 2 mg/kg: UNC-2 and 4 mg/kg: UNC-4) or vehicle were randomly assigned to Water-saline and Withdrawal-baclofen mice and IP administrated 30 min before WM testing. As shown in Figure 7A left, Water-Saline mice at UNC-4 dose exhibited significantly lower percentage of correct responses (46.3 ± 5.4%) compared with vehicle (71.1 ± 3.6%; *U* = 9, *p* = 0.0033), UNC-1 (77.1 ± 3%; *U* = 3, *p* = 0.0015) and UNC-2 (64.3 ± 4.3%; *U* = 12, *p* = 0.039). Figure 7A right shows that Withdrawal-Baclofen mice given UNC-4 dose also responded at chance (43.8 ± 6.2%) and significantly differed from vehicle (76.7 ± 5%; *U* = 7.5, *p* = 0.0039), UNC-1 (75 ± 5.7%; *U* = 4.5, *p* = 0.01) and UNC-2 (68.5 ± 5.2%; *U* = 9.5, *p* = 0.01). The analysis per block of 3 trials confirmed that Water-Saline (Figure 7B) and Withdrawal-Baclofen (Figure 7C) mice given the UNC-4 dose responded at chance on both blocks. Significant between-group differences were found for Withdrawal-Baclofen mice at UNC-4 dose and other groups on block A (versus vehicle: *U* = 16, *p* = 0.033; UNC-1: *U* = 7.5, *p* < 0.01; UNC-2: *U* = 9, *p* < 0.01). In addition, both Water-Saline and Withdrawal-Baclofen mice at UNC-4 dose significantly differed on block B from vehicle (*U* = 17, *p* = 0.022 and *U* = 10, *p* = 0.007, respectively) and UNC-1 (*U* = 10, *p* = 0.033 and *U* = 7, *p* = 0.028; respectively). The analysis per block also revealed that UNC-2 mice performed well on block A (Water-saline: 85.7 ± 6.7%; Withdrawal-baclofen: 77.8 ± 5.6%) but were impaired on block B (Water: 42.9 ± 9.25%; Withdrawal: 59.3 ± 7.4%). Significant differences were observed for Water-Saline mice between UNC-2 and vehicle (*U* = 12.5, *p* = 0.028) or UNC-1 (*U* = 7.5, *p* = 0.017) and for Withdrawal-Baclofen mice between UNC-2 or UNC-1 (*U* = 20, *p* = 0.04). Importantly, all groups significantly alternated above chance level when tested on trial 8 with a 5 s ITI (between 88.9 and 100%; *p* < 0.01 in all comparisons).

## 4 Discussion

The present study provides evidence that mice exposed to a 5-month voluntary alcohol intake displayed WM deficits along with significant decrease of global levels of repressive H3K9me^2^ in the PFC. The decrease of H3K9me^2^ occurred in concert with increased H3K9ac in the PFC and lasted up for 3 weeks after cessation of alcohol. After treatment with the GABA_B_ agonist baclofen during early withdrawal period, when WM was restored, the balance between H3K9me^2^ and H3K9ac was no longer disturbed in the PFC of withdrawal mice. Both marks were reduced after testing in the dHPC or remained unchanged in the amygdala and dorsal striatum, suggesting region-specific effects of alcohol withdrawal. Importantly, a significant and positive correlation existed between WM performance and the number of H3K9me^2^ positive neurons specifically in the PFC. Finally, we showed that acute pharmacological inhibition of G9a activity before WM testing impairs spontaneous alternation behavior in Withdrawal-Baclofen and Water-Saline mice. Collectively, our findings suggest that sustained decrease of prefrontal H3K9me^2^ may contribute, at least in part, to the long-lasting WM deficits observed several weeks after the cessation of chronic alcohol exposure.

In studies using animal models of human alcohol consumption, damages of the medial PFC neurons have been associated with deficits in various spatial WM tasks in both voluntary and non-voluntary models (Charlton and Perry, 2022). Indeed, all spatial WM tasks rely on the functional integrity of the medial PFC working in conjunction with the dHPC and, consistent with this, alcohol-induced damage of prefrontal neurons produced enduring WM impairments in rodents assessed through the spontaneous alternation T-maze or Y-maze tests (Vargas et al., 2014; Ikram et al., 2019). We previously reported that alcohol withdrawal after CAC produced long-lasting WM impairments in the T-maze spontaneous alternation (Dominguez et al., 2016) as well as long-lasting contextual memory deficits in a contextual and serial discrimination memory task in mice (Henkous et al., 2022). In the present study, the Withdrawal-saline mice exhibited significant WM deficits compared with Water-Saline controls. Specifically, Withdrawal-Saline mice showed no deficits on the 1st block of trials (Block A: Trials 1–4) but had low alternation rates on the 2nd block of trials (Block B: Trials 5–7), thus indicating that WM deficits resulted in a heightened vulnerability to proactive interference over the series -that is to say, an impaired capacity to suppress intentionally no-longer relevant information. However, Withdrawal-Saline mice showed no deficits during the pre-test trials using a delay of 30 s or during the post-test trial when the ITI was shortened from 90-s to 5-s (Trial 8), suggesting that alcohol withdrawal did not disturb attentional mechanisms and/or locomotor activity. Together, these observations confirmed and extended previous studies reporting impaired inhibition of proactive interferences in long-term abstinent alcoholic individuals (Noël et al., 2008; Chanraud et al., 2010). Systemic administration of the GABA_B_-receptor agonist baclofen has previously been shown to rescue spatial WM deficits using a similar T-maze spontaneous alternation task in several genetic mouse models (Gandal et al., 2012; Sinclair et al., 2017). Recently, we also showed corrective effects of repeated baclofen treatment during alcohol withdrawal on long-lasting spatial contextual memory deficits and excessive plasma and brain regional glucocorticoid levels in stressed withdrawal mice (Henkous et al., 2022). Confirming previous findings, we found that daily administration of baclofen during the 15 days of alcohol fading phase and early withdrawal reverses persistent WM impairments in withdrawal mice. Specifically, compared with Withdrawal-Saline animals, Withdrawal-Baclofen mice displayed high alternation scores at block B suggesting that baclofen improved animal’s capability to reset non-relevant information over the series of trials. We previously reported that baclofen administration reverses alcohol seeking behavior and rescues alterations in HPA axis activity in withdrawal mice pre-exposed to acute stress while having no effect in non-stressed mice (Rabat et al., 2019). However, some authors reported that baclofen decreases plasma cortisol levels in alcohol-dependent patients (Geisel et al., 2019) while others found no effect of baclofen on cortisol levels in alcoholic subjects (Farokhnia et al., 2018). Recently, Logge et al. (2023) proposed as a possible interpretation for the mechanism of action of baclofen to prevent alcohol use disorders may be through modulation of the stress-response systems rather than a direct anxiolytic effect of baclofen *per se*.

Abnormal regulation of epigenetic mechanisms has recently emerged as an important contributor to the long-term neural and behavioral changes associated with prolonged alcohol drinking and withdrawal (Bohnsack et al., 2018; Chen et al., 2019). Decreased levels of histone acetylation due to elevated histone deacetylases (HDAC) expression and activity were consistently observed in specific brain regions of animals exposed to excessive ethanol intake or protracted abstinence (Berkel and Pandey, 2017; Sharma et al., 2020; Bohnsack and Pandey, 2021). Previous studies of chronic intermittent ethanol exposure and withdrawal have demonstrated persistent over-representation of subsets groups of genes related to neurodevelopment and synaptic plasticity (such as *Bdnf*) and histone acetylation (such as *HDAC4* and *HDAC6*) and histone/DNA methylation specifically within the PFC and the hippocampus (Smith et al., 2016). While studies have investigated the role of histone H3K9me^2^ and G9a activity in neuroadaptations underlying sensitivity to drug reinforcement and drug-seeking behaviors (Maze et al., 2010; Sun et al., 2012; Anderson et al., 2018), limited literature exists regarding the role of H3K9me^2^ and G9a activity in neuroadaptations underlying alcohol-related behaviors. Recent work has suggested a role for G9a-mediated H3K9me^2^ mechanisms in anxiety-like disorders and stress-potentiated alcohol drinking (Berkel et al., 2018; Anderson et al., 2022). Interestingly, changes of H3K9 methylation marks and downregulation of G9a expression at the NR2B gene promoter were also associated with the long-lasting facilitation of N-methyl D-aspartate (NMDA) activity caused by chronic intermittent ethanol treatment and its removal (Qiang et al., 2011). In the present study, both Withdrawal-Saline and Withdrawal-Baclofen animals had reduced basal H3K9me^2^ levels in the PFC relative to Water-drinking mice. After WM testing, Withdrawal-Saline (impaired) mice exhibited a further decrease of prefrontal H3K9me^2^ which was not observed in Withdrawal-Baclofen (unimpaired) animals. Our results suggest that daily administration of the GABA_B_ receptor agonist baclofen during the alcohol fading phase and early withdrawal counteracts withdrawal-induced WM deficits through normalization of H3K9me^2^ in the PFC. In support of this, the magnitude of prefrontal H3K9me^2^ correlated positively with alternation performance when the four groups of mice were pooled together. In other brain regions, Withdrawal-Saline mice displayed either a decrease (dHPC) or no changes (amygdala and dorsal striatum) of H3K9me^2^, as compared with other groups, thus indicating that brain region-specific regulation of H3K9me^2^ by prolonged alcohol withdrawal. The present findings suggest that a sustained decrease of transcriptionally repressive H3K9me^2^ mark specifically in the PFC might be a critical component contributing to long-lasting WM impairments observed several weeks after the cessation of alcohol. However, since the mice were sacrificed 3 weeks after the cessation of alcohol, we therefore cannot assess whether the withdrawal-induced decrease of prefrontal H3K9me^2^ resulted from chronic alcohol exposure (and persisted into withdrawal) or from withdrawal *per se*.

The observed decrease of prefrontal H3K9me^2^ after a 3-week withdrawal period might suggest a more permissive chromatin environment allowing transcriptional de-repression of a subset of target genes. In this regard, we previously reported that long-lasting WM impairments correlated with a sustained increase of glucocorticoid (GC) response both during and after testing specifically in the PFC of withdrawal mice (Dominguez et al., 2018). Geisel et al. (2019) evidenced sustained increased GC levels in alcohol-dependent subjects, which decreased significantly in baclofen-treated patients, up to 14 weeks after abstinence. Among the target genes involved in post-translational regulation of GC function, the *GC* receptors themselves (GR encoded by NR3C1 gene) and the FK506 binding protein 51 (FKBP5, the direct negative feedback regulator of GR function), were found altered in the PFC and other cortico-limbic regions following CAC and alcohol withdrawal (McClintick et al., 2013; Vendruscolo et al., 2015; Gatta et al., 2021). Our results showing that Water-Saline mice displayed reduced H3K9me^2^ in the PFC are congruent with previous studies reporting decreased methylation of the FKBP5 gene in the PFC of adults with alcohol use disorders (Gatta et al., 2021; Lohoff et al., 2021). Since G9a-dependent H3K9me2 is required for DNA methylation (Poulard et al., 2021), these findings suggest that functional G9a–DNMT interactions are disturbed following prolonged alcohol exposure. These alterations of the FKBP5-GR loop can be counteracted by antidepressant treatment (Xing et al., 2015). Furthermore, findings from the FKBP5 gene in mice and FKBP5 genetic variants in humans have demonstrated an essential role for FKBP5 gene in modulating with alcohol withdrawal severity (Huang et al., 2014). Given that FKBP5 is a potent inhibitor of GR activation, it is possible that, at least in the PFC, decreased methylation of the FKBP5 gene following chronic alcohol consumption increases FKBP5 expression and reduces GR sensitivity to GC binding, leading to abnormal GC response and PFC dysfunction (Gatta et al., 2021).

Several studies have demonstrated that H3K9 lysine methyltransferase promoters (for example, G9a) dynamically interact with histone deacetylases to regulate functional interplay between histones acetylation and methylation states (Maze et al., 2010; Kennedy et al., 2013). The present data show that, as opposed to decreased H3K9me^2^, prefrontal H3K9ac was significantly increased after testing in Withdrawal-Saline mice relative to other (unimpaired) groups. Altogether, these findings suggest that, at least in the PFC, Withdrawal-associated WM deficits correlated with a shift in the balance between H3K9 acetylation (increased) and methylation (decreased) states towards a de-repression of downstream target genes. In agreement with our findings, chronic intermittent ethanol treatment and withdrawal induced an upregulation of the NMDA receptor 2B (NR2B) gene and this upregulation occurred through increased H3K9ac and concomitant decrease of G9a-mediated histone H3K9me^2^ in primary cortical neurons (Pian et al., 2010; Qiang et al., 2011). Our observation of daily administration of baclofen was able to improve WM and normalize H3K9 acetylation-methylation balance in the PFC agrees with previous reports reporting that GABA_B_ receptors influence NMDA receptors function and vice versa in physiological and pathological situations (Kantamneni, 2015). The increased expression levels of GluN2B proteins can be reversed by administration of baclofen in rats with diabetic neuropathic pain (Liu et al., 2014). Collectively, these findings support a role for histone H3 acetylation-methylation imbalance as a causal factor in the profound neuroadaptations that are apparent for several weeks after CAC in rodents.

Several preclinical studies demonstrated anxiety-related behavioral abnormalities and cognitive impairments associated with conditional G9a/GLP deficiency and pharmacological inhibition of G9a activity in the brain (Schaefer et al., 2009; Wang et al., 2018). Indeed, repeated IP injections of the G9a_inh_ UNC0642 were effective at reducing anxiety-like behaviors and reversing rapid tolerance to the anxiolytic effects of ethanol in a dose-dependent manner (Berkel et al., 2018). Chronic systemic administration of UNC0642 also suppressed stress-potentiated alcohol drinking when tested 2 weeks later whereas a single injection of UNC0642 was not sufficient for an anxiolytic effect measured 30 min later (Anderson et al., 2022). In our study, UNC0642 was injected at 1, 2, or 4 mg/Kg to Water-Saline and Withdrawal-Baclofen mice 30 min prior to the testing session, which is in line with the doses used in prior mouse studies (Kim et al., 2017; Anderson et al., 2022). Previous studies have reported that a single dose of UNC042 at 4 mg/Kg had no effect on anxiety-like behavior in C57BL/6 mice tested on elevated zero maze (Wang et al., 2018). In the current study, UNC0642 at the dose 4 mg/Kg (UNC-4) significantly decreased the rate of spontaneous alternation performances in both groups to levels comparable to those in Withdrawal-Saline mice. The analysis per block of 3 trials confirmed that UNC-4 mice were impaired at both blocks. Interestingly, the same analysis also revealed that UNC-2 mice displayed high alternation scores on the first block but performed at chance on the second one (at approximately 35–40 min after drug injection), suggesting that at this dose, the G9a inhibitor was able to increase vulnerability to proactive interference. Due to the route of administration in our study and the fact that we did not examine regional changes of H3K9me^2^ in the UNC-treated mice, we therefore cannot be certain that a 30 min-time interval between systemic drug injection and testing was sufficient to reduce H3K9me^2^ within defined brain regions, resulting in de-repression of target genes and WM impairments. Meanwhile, systemic UNC0642 administration can influence G9 activity in peripheral tissues and indirectly impact behavioral performances. Because G9a interacts directly with other transcriptional repressors or methylate a wide range of non-histone proteins, including G9a itself and histone deacetylase (HDAC1) (Poulard et al., 2021), it is also possible that G9a inhibition caused by acute UNC0642 injection disrupts the chromatin association of such complexes. Future studies are needed to explore the transcriptional and epigenetic mechanisms by which acute or repeated IP injections of UNC0642 produces brain region, cell-types and pathways-specific effects.

There are several limitations to this study that should be noted. First, only male animals were used in the current experiments and the above conclusions cannot be extrapolated to female. Previous studies have shown that sex- and gender-related differences in alcohol use and its consequences (Finn et al., 2010; Erol and Karpyak, 2015). Future studies are needed to investigate whether the present findings extend to female mice. Another major limitation is that we performed an immunohistochemical evaluation of global H3K9 acetylation and methylation patterns in the PFC and other brain regions. However, specific histone PTMs and DNA methylation patterns differ among tissues and cell types, and these differences contribute to establishing the epigenetic landscape of alcoholism (Krishnan et al., 2014). Also, although alcohol may have similar general effects on histone modifications across various tissues, it is likely that different cell types-specific expression profiles within a region orchestrate different regulatory programs. Indeed, G9a not only controls tissue-specific gene expression programs, but also influences neuronal subtypes identity within the same tissue (e.g., dopamine receptors D1 versus D2 in the striatum) (Maze et al., 2014). Future research is needed to gain a better insight into the epigenetic regulatory events affected during the cessation of alcohol after prolonged alcohol consumption in the brain.

In summary, the current study demonstrated that CAC followed by a 3-week alcohol withdrawal period leads to long-lasting WM impairments along with sustained decrease of H3K9me^2^ specifically in the PFC in male C57BL/6 mice. We showed that baclofen treatment during alcohol fading and early withdrawal phases effectively counteracts behavioral deficits and fully restored prefrontal H3K9me^2^ levels in withdrawal mice. Our study confirms and extends previous studies supporting a role for aberrant regulation of epigenetic mechanisms within specific brain regions in the long-term impairments of memory functions related to chronic alcohol exposure.

## Data availability statement

The raw data supporting the conclusions of this article will be made available by the authors, without undue reservation.

## Ethics statement

The animal study was approved by local ethical committee (CEE50, approval #24077) European Union Directive 2010/63/European Union guidelines for animal experiments. The study was conducted in accordance with the local legislation and institutional requirements.

## Author contributions

MC: Methodology, Writing – review & editing. MM: Conceptualization, Methodology, Writing – review & editing. NH: Methodology, Writing – original draft. Md’A: Methodology, Writing – review & editing. DB: Conceptualization, Resources, Supervision, Writing – original draft, Writing – review & editing. NM: Conceptualization, Funding acquisition, Supervision, Writing – original draft, Writing – review & editing.
